# Complete mitochondrial genome of the blister beetle *Hycleus scutellatus* Rosenhauer, 1856 (Coleoptera, Meloidae)

**DOI:** 10.1080/23802359.2022.2080603

**Published:** 2022-06-10

**Authors:** Pablo Mora, Eugenia E. Montiel, Teresa Palomeque, Pedro Lorite

**Affiliations:** Department of Experimental Biology, Genetics, University of Jaén, Jaén, Spain

**Keywords:** *Hycleus scutellatus*, blister beetle, mitogenome

## Abstract

In this study, we report the complete mitochondrial genome or mitogenome of the blister beetle *Hycleus scutellatus*, one endemic species from the Iberian Peninsula. The mitogenome was 16,035 base pairs in length, with an A + T content of 71.7%. It has 37 genes including 13 protein-coding genes, 22 transfer RNA genes and 2 ribosomal RNA genes. To analyze the evolutionary position of *H. scutellatus*, we constructed a phylogenetic tree using all available mitogenomes from species of the family Meloidae. The results show that *Hycleus* species are very close to the genus *Mylabris*. We present here the mitogenome of *H. scutellatus* as a new resource to elucidate the phylogenetic relations among the Meloidea family, being this source very useful for future evolutionary analyses of blister beetles.

The family Meloidae, the blister beetles, contains about 3000 species placed in about 130 genera (Riccieri et al. 2020). The name of ‘blister’ remains into the ability of these insects to secrete cantharidin, a chemical fluid that causes irritation and blistering when it contacts with animal skins. Males produce this substance not only as a defensive tool but also as a copulatory gift for females during the mating process (Moed et al. 2001). There is a recent interest in the blister beetles due to the anticancer properties of the cantharidin and its derivatives (Naz et al. 2020). One of the most speciose genera of the family is *Hycleus.* Here we present the complete mitogenome of *Hycleus scutellatus* Rosenhauer, 1856, one endemic species from the Iberian Peninsula (Trotta-Moreu and García-París 2001). Genetic information on this species is scarce and is limited to a cytogenetic study that included the analysis of a repetitive DNA family (Ruiz-Torres et al. 2021). Data from mitogenomes can be useful for future phylogenetic studies in this beetle group.

The specimens of *H. scutellatus* were collected in Alcudia de Guadix, Spain (37.79 N, −3.78 W). *H. scutellatus* is not an endangered or protected species thus we did not need any specific permission for its collection. Specimens of the analyzed population were stored in the RNM-924 Group collection (voucher 2006_HS_01; contact email: plorite@ujaen.es) in the University of Jaén, Spain. Genomic DNA was isolated using the NucleoSpin Tissue kit (Machery-Nagel GmbH & Co). Genomic DNA was used for the construction of a library with 750 bp fragments and sequenced using the HiSeq 2000 Illumina platform, with pair-ends reads (101 bp each) at Macrogen Japan Corp. (Tokyo, Japan). Graph-based clustering analysis was performed on RepeatExplorer, implemented within the Galaxy environment (http://repeatexplorer.org/) (Novák et al. 2010, 2013). Sequences corresponding to mtDNA were selected and assembled with CAP3 software using the *Hycleus cichorii* mitogenome (Wu et al. 2018) as reference. Two regions were not recovered using genome sequencing. These two gaps were closed by PCR amplification using a two pair of primers; ND1-F (TGAATTGGAGCCCGGCCAGCAGAA) and ND1-R (GTAGCGTTTACTACTTTAATGGAGCG) for the first region and RControl-F (ATAATGGGGTCTCTAATCCCAGT) and the ARNt-Met primers (AGGGTATGAACCTAAWAGC) for the second one. The mitogenome was annotated using MITOS (http://mitos.bioinf.uni-leipzig.de/help.py) (Bernt et al. 2013) with manual curation for consistent start/stop codons. The complete mitogenome of *H. scutellatus* was 16,035 base pairs in length, being comparable to other blister beetles. The A + T content was high (71.7%) as a typical feature of insects. The mitogenome contained the typical 13 protein-coding genes (PCGs), two rRNAs genes and 22 tRNAs. All PCGs used typical ATN start codons: ATG (*atp6*, *coIII*, *nd4* and *cytb*), ATT (*nd2*, *coI*, *atp8*, *nd3* and *nd4L*), ATA (*nd5*, *nd6* and *nd1*) or ATC (*coII*). Eight out of the 13 PCGs have the TAA stop codon and one of them (*nd3*) the TAG stop codon. Four PCGs have incomplete T stop codons (*coI*, *coII*, *nd5* and *nd4*). All tRNAs could be folded into the typical secondary structure, except for the *tRNA-Ser* (AGN), which lacks the DHU arm. The length of *rrnL* was 1,282 bp and the length of *rrnS* was 733 bp.

Complete mitogenomes from other species from the family Meloidae were recovered from GenBank and used for phylogenetic analysis (Figure 1). Sequences from the 13 PGCs were aligned using MAFFT and concatenated for phylogenetic analysis. The phylogenetic relationships were reconstructed using the Maximum-Likelihood method (Saitou and Nei 1987) implemented in the program MEGA version 11 (Tamura et al. 2021). GTR + G + I was selected as the best model as it showed the lowest BIC (Bayesian Information Criterion) value. The nodes were assessed with 1000 bootstrap replicates. Phylogenetic analysis showed that *Hycleus* and *Mylabris* species appear clustered together in a very well-supported clade, which is in agreement with other studies that recovered the tribe Mylabrini as a monophyletic group (Bologna and Pinto 2001; Du et al. 2017). However, the species of both genera do not appear separately within the clade. Separation of *Mylabris* and *Hycleus* received strong support in other phylogenetic studies based on the 16S and ITS2 sequences (Bologna et al. 2005, 2008), although these studies included a limited number of species. *Mylabris* and *Hycleus* are the most speciose genera in the Meloidae family, their phylogenetic relationships are still understudied and some authors have highlighted the need of more phylogenetic analyses within the group (Pan and Bologna 2014; Salvi et al. 2019; Riccieri et al. 2020).

**Figure 1.**
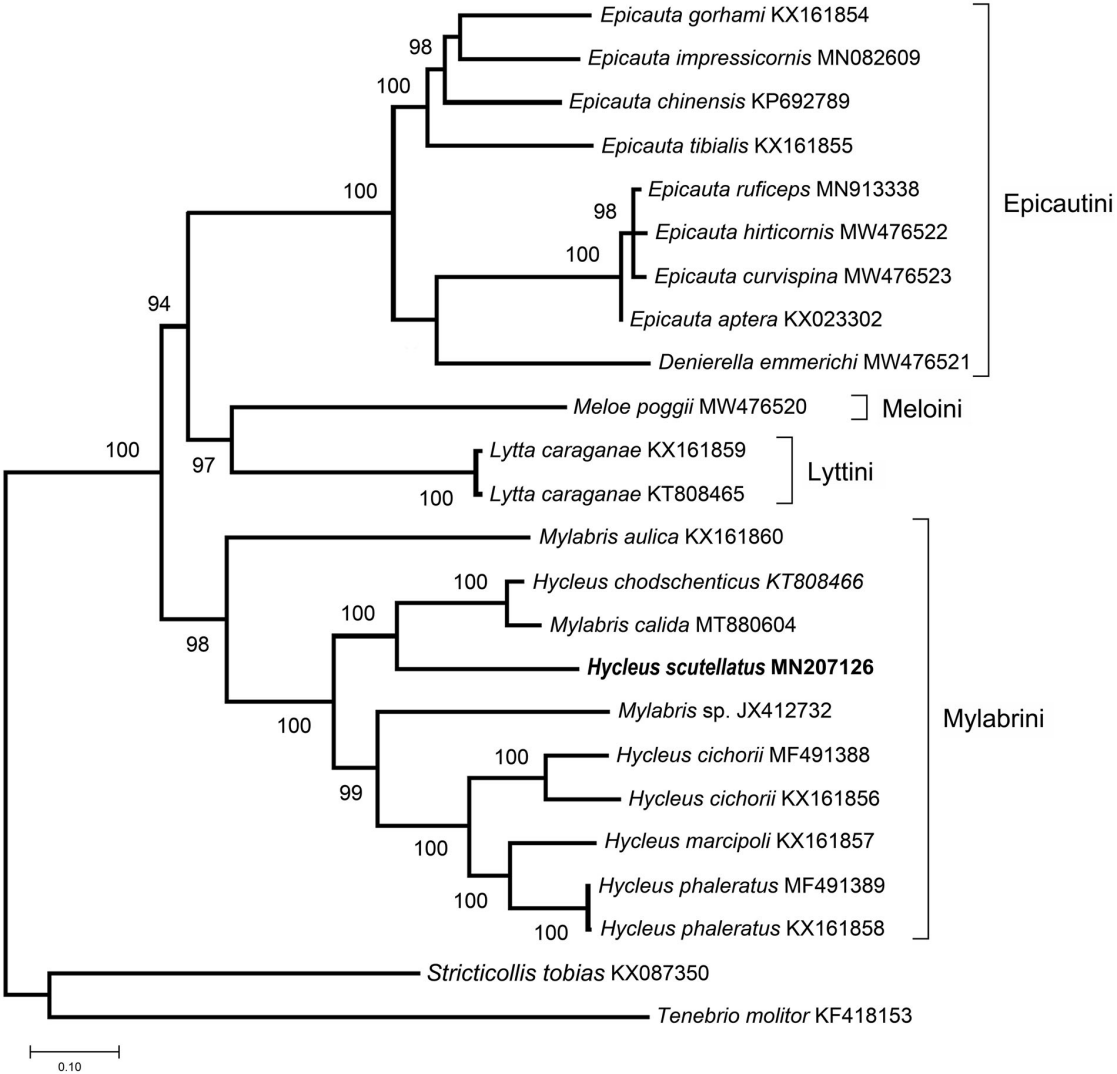
Maximum-likehood phylogenetic tree using the 13 PCGs concatenated from all the available datasets of the family Meloidae and two species of related families used as outgroup: *Tenebrio molitor* and *Stricticollis tobias*. Only bootstrap support values greater than 70% are shown.

## Data Availability

The genome sequence data that support the findings of this study are openly available in GenBank of NCBI at [https://www.ncbi.nlm.nih.gov] under the accession no. MN207126. The associated BioProject, SRA, and Bio-Sample numbers are PRJNA791791, SRR17319087, and SAMN24345222 respectively.
